# Chitin-Glucan Complex Hydrogels: Optimization of Gel Formation and Demonstration of Drug Loading and Release Ability

**DOI:** 10.3390/polym14040785

**Published:** 2022-02-17

**Authors:** Diana Araújo, Thomas Rodrigues, Vítor D. Alves, Filomena Freitas

**Affiliations:** 1 Associate Laboratory i4HB, School of Science and Technology, Institute for Health and Bioeconomy, NOVA University Lisbon, 2819-516 Caparica, Portugal; df.araujo@campus.fct.unl.pt (D.A.); ta.rodrigues@campus.fct.unl.pt (T.R.); 2 UCIBIO, Applied Molecular Biosciences Unit, Department of Chemistry, School of Science and Technology, NOVA University Lisbon, 2819-516 Caparica, Portugal; 3 LEAF, Linking Landscape, Environment, Agriculture and Food Research Center, Laboratório Associado TERRA, Instituto Superior de Agronomia, Universidade de Lisboa, Tapada da Ajuda, 1349-017 Lisboa, Portugal; vitoralves@isa.ulisboa.pt

**Keywords:** hydrogels, chitin-glucan complex, freeze–thaw cycles, swelling ratio, caffeine, drug delivery

## Abstract

Chitin-glucan complex (CGC) hydrogels were fabricated through a freeze–thaw procedure for biopolymer dissolution in NaOH 5 mol/L, followed by a dialysis step to promote gelation. Compared to a previously reported methodology that included four freeze–thaw cycles, reducing the number of cycles to one had no significant impact on the hydrogels’ formation, as well as reducing the total freezing time from 48 to 18 h. The optimized CGC hydrogels exhibited a high and nearly spontaneous swelling ratio (2528 ± 68%) and a water retention capacity of 55 ± 3%, after 2 h incubation in water, at 37 °C. Upon loading with caffeine as a model drug, an enhancement of the mechanical and rheological properties of the hydrogels was achieved. In particular, the compressive modulus was improved from 23.0 ± 0.89 to 120.0 ± 61.64 kPa and the storage modulus increased from 149.9 ± 9.8 to 315.0 ± 76.7 kPa. Although the release profile of caffeine was similar in PBS and NaCl 0.9% solutions, the release rate was influenced by the solutions’ pH and ionic strength, being faster in the NaCl solution. These results highlight the potential of CGC based hydrogels as promising structures to be used as drug delivery devices in biomedical applications.

## 1. Introduction

Hydrogels are three-dimensional network structures fabricated from synthetic or natural polymers capable of absorbing large amounts of water [1,2,3]. Biopolymer hydrogels have attracted increasing interest due to their biocompatibility, biodegradability, environmentally friendly features, and tissue-mimicking consistency. These valuable characteristics make them suitable materials for utilization in a wide range of applications from food and agriculture [4] to cosmetics [5] and biomedicine [6].

Depending on the method used to crosslink the polymer chains, hydrogels can be classified as chemical or physical. Chemical hydrogels are mostly connected through a covalently cross-linked network, in which the addition of crosslinking agents promotes the reaction between the functional groups of the polymer chains [7,8]. However, those chemical agents are often toxic compounds, and their presence may have adverse effects, such as undesirable reactions with bioactive substances or affect the hydrogels’ biocompatibility [9]. On the other hand, physical hydrogels are obtained by crosslinking the polymer chains through non-covalent interactions such as ionic interactions, hydrogen bonds, chain entanglements, van der Waals forces, or hydrophobic interactions [8]. Therefore, physically crosslinked hydrogels, especially biopolymer-based ones, are promising materials for use in the biomedical field due to the use of mild conditions during their fabrication, and the absence of organic solvents and toxic crosslinking agents [10,11].

The water insoluble and highly hydrophilic biopolymer chitin-glucan complex (CGC) is the main component of the inner cell wall of yeast and fungi, composed of N-acetyl-glucosamine and glucose monomers [12,13,14]. Owing to its biocompatibility and biodegradability characteristics—along with intrinsic antioxidant, anti-inflammatory, and antibacterial properties [15,16]—CGC has been applied as a food additive [17], an anticholesterol agent [18], and for wound healing [19]. Nevertheless, due to the numerous hydrogen bonds between its polymeric chains, similarly to chitin and others chitin-derived polymers, CGC is insoluble in the most common solvents [3,15]. Recently, alkali solvents based on NaOH or KOH have emerged as alternative solvents systems for CGC dissolution, through the freeze–thaw method [20]. In this process, the presence of a hydrated alkali component, below the freezing point, promotes the disruption of the polymer chain matrix by breaking inter and intramolecular hydrogen bonds, allowing for polymer dissolution [21]. CGC based physical hydrogels can be obtained by dialyzing the CGC dissolved in the alkali systems. During the dialysis process, gelation is induced by the interactions established between the CGC molecules promoted by the reduction of the ionic forces [22].

In this study, the impact of the number of freeze–thaw cycles and the freezing time during the procedure on the hydrogel-forming capacity of CGC was evaluated in terms of hydrogels’ chemical composition, morphology, and mechanical properties. The optimized CGC hydrogel was characterized as to its rheology, swelling properties, drug loading, and drug release capacity.

## 2. Materials and Methods

### 2.1. Materials

Yeast biomass was obtained by cultivation of the yeast *Komagataella pastoris* (DSM 70877) using glycerol as the sole carbon source, as described by Farinha et al. [12]. CGC was extracted from *K. pastoris* biomass by the hot alkaline procedure described by Araújo et al. [23] and it represented 20 wt% of the cell dry mass. CGC presented a chitin content of 35.6% and a degree of acetylation (DA) of 63.4%.

### 2.2. Preparation of CGC Hydrogels

The CGC hydrogels were prepared as described by Araújo et al. [22], with slight modifications. Briefly, the CGC powder (0.5 g) was dispersed in a NaOH 5 mol/L solution (25 g), and the suspensions were kept at −20 °C, for either 18 or 48 h. During that period, different number of freeze–thaw cycles (0, 1, 2, or 3) were performed, being the thawed suspensions extensively stirred (500 rpm, 1 h), at room temperature in each cycle. After centrifugation (20,000× *g*, 30 min, 4 °C) to eliminate the undissolved material, the hydrogels were prepared by dialyzing the soluble fractions with a 12–14 kDa MWCO membrane (Spectra/Por^®^, Spectrum Laboratories Inc., Piscataway, NJ, USA), in deionized water, at room temperature, for 48 h. The obtained hydrogels were labelled according to the solvent system used (NaOH 5 mol/L) and the number of freeze–thaw cycles performed. The hydrogels prepared by freezing during 48 h using 0, 1, 2, or 3 freeze–thaw cycles were identified as Na5_0_, Na5_1_, Na5_2_, and Na5_3_ hydrogels, respectively, while the hydrogel prepared by 1 freezing cycle of 18 h was coded as Na5_1_* hydrogel (Table 1).

### 2.3. CGC Hydrogels Characterization

#### 2.3.1. Chemical Characterization

The water content of the hydrogels was assessed gravimetrically by freeze drying the hydrogel samples, using the equation

Water content = ((W_wet_ − W_dry_)/W_wet_) × 100 (1)

where W_dry_ (g) represents the dry mass of a pre-weighed amount of the hydrogel (W_wet_, g).

The chitin content and the degree of acetylation were determined by elemental analysis as described by Araújo et al. [20].

#### 2.3.2. Morphology, Density, and Porosity

The structure and morphology of the CGC hydrogels were characterized by scanning electron microscopy (SEM). The hydrogels were analyzed with a TM3030 tabletop microscope (Hitachi in High Technologies, U.S.) equipped with a sample holder with refrigeration. Hydrogels’ samples were observed at a temperature of −4 °C, using a magnification of 500×.

The density (ρ, g/cm^3^) of the freeze-dried CGC hydrogels was determined by equation

ρ = W_dry_/V_dry_ (2)

where W_dry_ and V_dry_ represent the weight (g) and volume (cm^3^) of the hydrogel, respectively.

The porosity of the CGC hydrogels was determined using the solvent replacement method [24]. Pre-weighed freeze-dried CGC hydrogels (W_0_, g) were immersed in absolute ethanol, for 30 min, in sealed tubes. After 30 min, excess ethanol on the surface was blotted and the samples were weighed. The porosity was calculated using the equation

Porosity (%) = (W_30_ − W_0_)/ρV_T_ (3)

where W_30_ and W_0_ represent the hydrogel weight (g) at 30 min and 0 min, respectively, ρ is the density of ethanol (0.790 g/cm^3^) and V_T_ (cm^3^) is the total volume of the hydrogel sample.

### 2.4. Compressive Mechanical Analysis

The compressive mechanical properties of the CGC hydrogels were assessed with a texture analyzer TMS-Pro (Food Technology Corporation, England, UK) equipped with a 50 N load cell. Cylindrical hydrogels samples in the wet state (13.8 cm diameter, 0.7–1.1 cm height) were subjected to a compression of up to 80% strain of the samples original weight, at a speed rate of 60 mm/min, using an aluminum plunger with 60 mm diameter. The maximum tension of the compression corresponds to the hardness (kPa) and the toughness (kJ/m^3^) was calculated by measuring the area underneath the stress–strain curve of each sample. Compressive modulus (kPa) was obtained as the slope of initial linear region. All the experiments were performed at room temperature (20 ± 0.2 °C).

### 2.5. Rheological Properties

The rheological properties of the Na5_1_* and Na5_1_* loaded hydrogels were analyzed using a controlled stress rheometer (HAAKE MARS III, Waltham, MA, USA Thermo Scientific), equipped with a plate–plate serrated geometry (diameter 20 mm) with a 1.5 mm gap. Hydrogel samples with a similar thickness (~3 mm) were equilibrated at 25 ± 0.03 °C, for 5 min. The viscoelastic properties were determined by applying frequency sweeps at a constant tension within the linear viscoelastic region, for a frequency range from 0.01 to 1 Hz.

### 2.6. Swelling and Water Retention Behavior

To assess the swelling properties, pre-weighed cylindrical freeze-dried samples of the Na5_1_* hydrogel were immersed in deionized water, NaCl 0.9% or phosphate buffered saline (PBS), at 37 °C. At different time intervals, samples were carefully taken out from the solutions, blotted with a filter paper, and weighed (W_wet_, g). The swelling ratio was determined using the equation

Swelling ratio (g/g) = (W_wet_ − W_dry_)/W_dry_ (4)

where W_dry_ (g) represents the initial mass of dry hydrogel.

To evaluate the water retention behavior of the structures, the equilibrated hydrogels were taken out from the solutions and weighed (W_e_), after blotted with a tissue paper. Swollen hydrogels were incubated at 37 °C and weighed (W_t_) over time. Water retention was calculated by the equation

Water retention (%) = (W_t_/W_e_) × 100 (5)

### 2.7. Drug Loading

Caffeine (Alfa Aesar, 99%) was used as model drug to assess drug loading and drug release behavior of the Na5_1_* hydrogels. For drug loading, pre-weighed cylindrical freeze-dried hydrogel samples were immersed in a caffeine solution (1.0 wt%), for 24 h, at room temperature. After that period, the loaded hydrogels’ samples were carefully taken out from the solution, blotted with a filter paper, and weighed (W_L_, g). Drug loading (DL, g) was determined by the equation

DL (g) = (W_L_ − W_dry_) × C_caf_ (6)

where W_dry_ (g) represents the initial mass of dry hydrogel and C_caf_ (wt%) corresponds to the concentration of caffeine solution.

The entrapment efficiency (EE, %) of caffeine in the hydrogels was calculated using the equation

EE (%) = (DL/W_caf_) × 100 (7)

where W_caf_ (g) represents the mass of caffeine.

### 2.8. Characterization of the Loaded Hydrogels

The Na5_1_* hydrogels and the caffeine loaded Na5_1_* hydrogels were characterized by Fourier-transform infrared spectroscopy (FT-IR). The analysis was conducted with a Spectrum II spectrometer (Perkin-Elmer, Llantrisant, UK) and the spectra were obtained between 500 and 4000 cm^−1^ after 10 scans, at room temperature.

The mechanical and rheological properties of Na5_1_* loaded hydrogels were assessed as described in Section 2.4 and Section 2.5, respectively.

### 2.9. In Vitro Drug Release Studies

The freeze-dried Na5_1_* loaded hydrogels’ samples were immersed in 100 mL of different physiological media: PBS (pH 7.4) and NaCl 0.9% (pH 5.5), at 37 °C, for 3 h, under constant stirring (100 rpm). Periodically, 2 mL of the release medium were withdrawn, and 2 mL of fresh medium, preheated at 37 °C, were added to keep the volume of the solution constant. The caffeine concentration in the withdraw solution was determined by UV–vis spectrophotometer (CamSpec M509T, Leeds, UK) at a wavelength of 273 nm [25], for a concentration range of 0.16–10 mg/L. Caffeine release was obtained by the equation

Caffeine release (%) = ((C_w_ × V)/DL) × 100 (8)

where C_w_ (g/L) is the caffeine concentration in the withdraw solution, V (L) is the volume of the release media, and DL (g) is the amount of loaded drug. The caffeine cumulative release was fitted to the Korsmeyer–Peppas model [26].

### 2.10. Statistical Analysis

The experimental data from all the studies were analyzed and the results were expressed as mean ± standard deviation (SD). Error bars represent the standard deviation (*n* ≥ 3).

## 3. Results

### 3.1. Hydrogels Formation

The freeze–thaw procedure followed by dialysis, recently reported by Araújo et al. [22], was used to dissolve CGC in NaOH 5 mol/L (freezing at −20 °C, 4 freeze–thaw cycles, 48 h total freezing time) and prepare CGC hydrogels (labelled as Na5 hydrogels), which exhibited a dense and stiff gel structure. Following those results, the present study aimed at optimizing the procedure by assessing the impact of reducing the number of freeze–thaw cycles and of the freezing time on the hydrogels’ properties.

Firstly, the effect of the number of cycles was studied by applying 1, 2, or 3 freeze–thaw cycles (samples Na5_1_, Na5_2_, and Na5_3_, respectively) (Table 1). The procedures’ performance was compared to that previously reported for four cycles. An experiment with no freeze–thaw cycles (sample Na5_0_), in which CGC was simply contacted with the NaOH solution (at room temperature, for 48 h), was also performed for comparison. In this case, a viscous slurry was formed, with very low CGC dissolution, and no hydrogel formation upon dialyzing the supernatant recovered from the mixture. This outcome may be due to the non-deacetylation of chitin that the freeze–thaw procedure induces [20], and consequently, the low dissolution of CGC in the solvent system.

**Figure 1:**
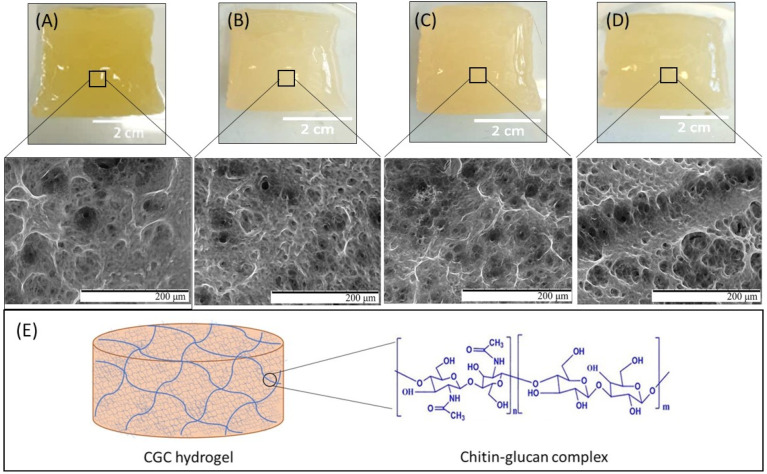
Macroscopic aspect and SEM images of Na5_1_ (**A**), Na5_2_ (**B**), Na5_3_ (**C**), and Na5_1_* (**D**) hydrogels under magnification 500×; chemical structure of CGC and schematic representation of the CGC hydrogel (**E**).

Except for Na5_0_, all CGC solutions in NaOH 5 mol/L formed hydrogels upon coagulation by dialysis (Figure 1). For the same freezing time (48 h), increasing the number of freeze–thaw cycles, from 1 to 3 cycles, led to a slight decrease in the polymer’s content (from 1.68 ± 0.17 to 1.42 ± 0.02 wt%, respectively). These results show that a single freeze–thaw cycle is sufficient for CGC gelling and the obtained hydrogels have a higher polymer content. Subsequently, the experiment was repeated for 1 freeze–thaw cycle, but the freezing time was reduced from 48 to 18 h (sample Na5_1_*). The Na5_1_ and Na5_1_* hydrogels, both prepared with 1 freeze–thaw cycle, presented similar polymer content (1.68 ± 0.17 and 1.66 ± 0.11 wt%, respectively), indicating that freezing time had no significant impact on CGC dissolution in the NaOH solvent system.

These results show that the procedure for CGC gelling can be simplified by reducing both the number of freeze–thaw cycle and the total freezing time. All the resulting hydrogels were characterized to evaluate their physicochemical properties and select the most suitable procedure for yielding structures with superior performance.

### 3.2. Chemical Characterization of the Hydrogels

Table 1 shows the chemical characterization of the CGC hydrogels. It can be observed that all hydrogels exhibited a water content above 97%, characteristic of these structures. No sodium was detected in the hydrogels, which demonstrates the efficient removal of the ion from the structure during the dialysis process.

As shown in Table 1, the chitin content was similar for all CGC hydrogels, however a slight decreasing was observed as the freezing cycles increased. In fact, the Na5_1_ hydrogels presented a chitin content of 25.63 ± 0.78%, while the Na5_3_ hydrogels shown 23.85 ± 0.14% of chitin. Moreover, the Na5_1_* hydrogels exhibited the lowest chitin content (21.51 ± 1.49%), suggesting that extended freezing time might be required to dissolve the enriched chitin CGC macromolecules in the NaOH solution.

### 3.3. Morphological Characterization

As shown in Figure 1, all CGC hydrogels were translucid, presented a yellow coloration and their shape was molded by the dialysis tubing. Furthermore, despite the different approaches applied for their preparation, all hydrogels exhibit similar macroscopic characteristics.

The morphological features of the CGC hydrogels were evaluated by SEM analysis (Figure 1). Similarly to the Na5 hydrogels [22], all structures presented a heterogeneous, compact, and dense three-dimensional network microstructure made of polymeric chains. The porous structures of the obtained hydrogels seem to be slightly affected by the number of freeze–thaw cycles, with the pore size increasing as the number of cycles increases. As shown in Figure 1, the Na5_3_ hydrogels exhibited a microstructure composed by larger pores when compared to Na5_1_ hydrogels. This fact might be explained by the lower polymer concentration present in the Na5_3_ hydrogels (Table 1). It has been reported that pore volume and pore size distribution are affected by the polymer content present during hydrogel formation [27]. Indeed, the increasing intermolecular crosslinks and physical entanglements present in hydrogels with high polymer concentrations leads to the formation of smaller pore volumes and pore sizes. Similar results were reported for chitin hydrogels [28], where the average pore size decreased from 8 to 5 µm in diameter as the chitin concentration decreased from 1 to 2 wt%.

Additionally, the SEM micrographs demonstrated that reducing the freezing time affected the hydrogels’ microstructure (Figure 1). Despite the similar polymer content of the Na5_1_ and Na5_1_* hydrogels (1.68 ± 0.17 and 1.66 ± 0.11 wt%, respectively), the lower freezing time (18 h) applied during preparation of the Na5_1_* hydrogels induced the formation of microstructures with larger pores. Analogous results were reported by Figueroa-Pizano et al. [29] for chitosan-poly(vinyl alcohol) hydrogels, where those produced with 4 h of freezing time presented larger pores than those formed with 12 h of freezing time.

### 3.4. Hydrogels’ Porosity and Density

**Figure 2:**
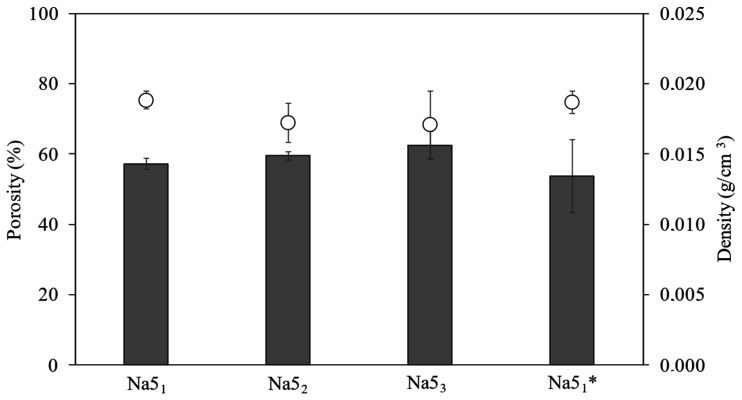
Porosity (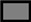) and density (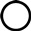) of the CGC hydrogels.

The porosity and density of the hydrogels are significantly dependent on their morphological characteristics and are important parameters for controlling the hydrogels’ physicochemical properties and kinetics of drug release [24]. As shown in Figure 2, the porosity values were similar for all CGC hydrogels, ranging from 53.8 ± 10.3 to 62.6 ± 3.9%. Even so, the Na5_3_ hydrogels presented the highest porosity value (62.6 ± 3.9%) which suggests that the porosity might have increased with increasing number of freeze–thaw cycles. Porosity depends on the size and number of pores per unit of volume. From the SEM micrographs, the pore size is easier to observe, and the structure of the Na5_3_ hydrogels comprised larger pores than Na5_1_ and Na5_2_ (Figure 1), which is consistent with the higher porosity measured. On the other hand, the Na5_1_* hydrogels exhibited the lowest porosity values (53.8 ± 10.3%), demonstrating that reducing the freezing time might have induced a decrease in the hydrogels’ porosity. Overall, the prepared CGC hydrogels exhibited interesting porosity levels that render them suitable for application in areas such as drug delivery [30] and/or tissue engineering [31].

The density of the CGC hydrogels is shown in Figure 2. It can be observed that the density of the hydrogels slightly decreased with the number of freeze–thaw cycles. Thus, the Na5_1_ and Na5_1_* hydrogels exhibited similar density values (0.019 ± 0.001 g/cm^3^) and the highest ones. These results are consistent with the higher polymer content present in such hydrogels (Table 1), and the resulting increase in the degree of crosslinking observed in the SEM micrographs (Figure 1). Hence, the denser CGC hydrogels (Na5_1_ and Na5_1_*) showed the lowest porosity, similar to the results reported for several polymer-based hydrogels, including chitosan [24] and collagen hydrogels [31].

### 3.5. Mechanical Properties

**Figure 3:**
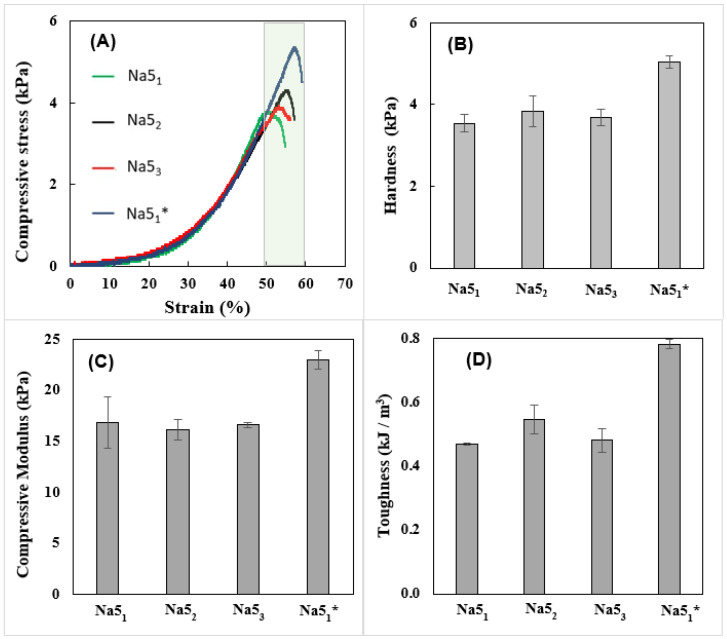
Compressive mechanical properties of the CGC hydrogels: compressive stress (**A**) stress–strain curves, hardness (**B**), compressive modulus (**C**), and toughness (**D**).

The mechanical properties of the CGC hydrogels (Figure 3)—namely, their hardness, compressive modulus, and toughness—were obtained by applying a single compression (80% of the initial height) to wet CGC hydrogel samples. As shown in Figure 3, the mechanical characteristics of the CGC hydrogels were not significantly influenced by the number of freeze–thaw cycles. The compressive stress–strain curves of the CGC hydrogels are represented in Figure 3A. The maximum compressive stress obtained represents the force required to produce the deformation of the hydrogels and corresponds to the hardness value (Figure 3B). It can be observed that similar stress–strain profiles were obtained for all hydrogels, with rupture strain occurring between 50% and 60% for compressive stress values of 80% (Figure 3A). Additionally, identical hardness values were achieved for the Na5_1_, Na5_2_ and Na5_3_ hydrogels (3.55 ± 0.23, 3.85 ± 0.38 and 3.69 ± 0.21 kPa, respectively), while higher values were presented by the Na5_1_* hydrogels (5.04 ± 0.14 kPa). The hardness values obtained were lower than those previously reported for the CGC Na5 hydrogels (7.23 ± 0.78 kPa) [22]. This difference is mainly related to the higher polymer content of those hydrogels (2.28 wt%), which increased the crosslinking between the polymer chains and, consequently, improved the hydrogel’s mechanical properties [32].

The compressive modulus and toughness of the CGC hydrogels, obtained from the stress–strain curves, are represented in Figure 3C,D, respectively. As shown in Figure 3C, similar compressive moduli were displayed by the Na5_1_, Na5_2_, and Na5_3_ hydrogels (16.8 ± 2.6, 16.2 ± 1.0, and 16.6 ± 0.3 kPa, respectively), demonstrating that the number of freezing cycles had no significant impact on their stiffness. Nonetheless, the Na5_1_* hydrogels were the stiffest material, characterized by a considerably higher compressive modulus (23.0 ± 0.89 kPa). As expected, analogous behavior was obtained for the hydrogels’ toughness values. Figure 3D shows that the Na5_1_* hydrogels exhibited the highest toughness value (0.78 ± 0.015 kPa), while the remaining hydrogel samples displayed values between 0.47 ± 0.003 and 0.55 ± 0.045 kPa. Despite the similar polymer content, the wispy porous structure of the Na5_1_* hydrogels might have resulted in higher strength, thus improving their compressive modulus.

Given these results, the Na5_1_* CGC hydrogels were selected for further characterization, including rheological properties, swelling behavior, and drug delivery capability.

### 3.6. Rheological Properties

**Figure 4:**
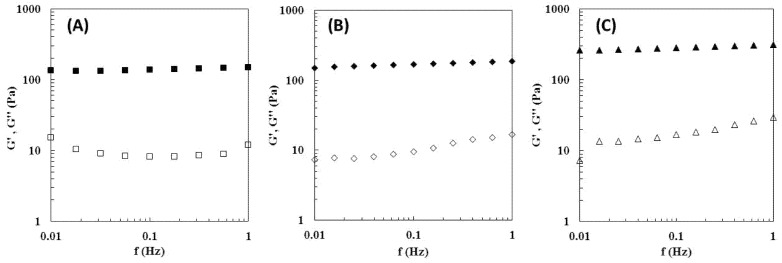
Rheological properties of the Na5_1_* hydrogel (**A**), Na5_1_* rehydrated hydrogel (**B**) and Na5_1_* loaded hydrogel (**C**), at 25 °C. Mechanical spectrum storage (G′, solid symbols) and loss moduli (G″, open symbols).

The viscoelastic properties of the Na5_1_* hydrogels (Figure 4A) show that the storage modulus (G′) displayed values one order of magnitude higher than the loss modulus (G″) over the entire range of frequencies, characteristic of their solid-like nature [22]. This behavior also indicates that the hydrogels exhibited predominately elastic characteristics [33]. Moreover, both dynamic moduli are independent of the frequency, thus revealing the formation of a stable gel [34]. Analogous behavior and similar G′ and G″ values (~100 and ~10 Pa, respectively) were obtained for hydrogels prepared with a chitin nanofiber content of 0.4 wt%, in the same range of frequency [35]. Recently, Ferreira et al. [36] also described an identical profile for gels prepared with *Aspergillus niger* CGC dissolved in ionic liquids (ILs). However, those gels presented higher values of both dynamic moduli which might be explained by the presence of ILs in the gel structure [36].

A similar profile was reported for the CGC Na5 hydrogels [22], however this hydrogel presented significantly higher values for both G′ and G″. In particular, for the same frequency (0.1 Hz), the Na5_1_* hydrogel presented a G′ of 136.8 ± 11.1 Pa, while a higher value (389 Pa) was found for the Na5 hydrogel. This fact is explained by the increased polymer content of the Na5 hydrogels (2.28 wt%) compared to the Na5_1_* hydrogels (1.66 wt%), which directly improve their mechanical properties.

### 3.7. Swelling Behavior and Water Retention Kinetics

**Figure 5:**
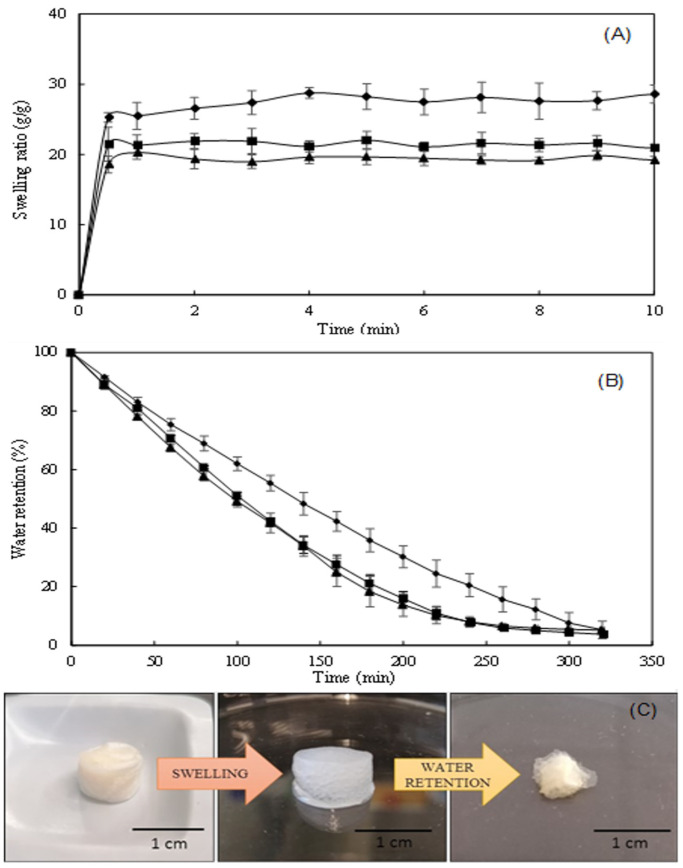
Swelling behavior (**A**) of Na5_1_* hydrogels in PBS (■), NaCl 0.9% (▲) and deionized water (◆), water retention kinetics (**B**) of water-swollen hydrogels, at 37 °C and macroscopic aspect of hydrogels (**C**) after the two processes.

The hydrogels’ water absorption capacity (swelling behavior) directly affects their drug loading and delivery capability [37]. The swelling ratio of the CGC hydrogels in the different tested media (PBS, NaCl 0.9%, and deionized water) at 37 °C is shown in Figure 5A. For the three tested media, the Na5_1_* hydrogels displayed excellent water absorption capacity, reaching the swelling equilibrium immediately (less than 1 min). This can be explained by the high hydrophilicity of CGC macromolecules that possess numerous hydrophilic groups, namely, hydroxyl and amino groups, capable of establishing hydrogen bonds with the water molecules [15]. It can be noticed that the swelling capacity of the Na5_1_* hydrogel was slightly higher in deionized water than in the other tested media (Figure 5A). Indeed, after 30 s, the hydrogel placed in water reached a swelling ratio of 25.3 ± 0.28 g/g, while lower values were obtained in PBS and NaCl 0.9% (21.5 ± 2.42 and 18.6 ± 1.20 g/g, respectively), for the same time period. This fact is probably related to the higher ionic strength of the PBS and NaCl 0.9% solutions, which decreases the swelling ability of polyelectrolyte hydrogels, due to the decreased osmotic pressure difference between the hydrogel structure and the solution [38].

Significantly lower values were reported by Wu et al. [39] and Udeni Gunathilake et al. [40] for chitin-based hydrogels (~6 g/g) and chitosan-based hydrogels (4.1 g/g), respectively. In fact, the nearly spontaneous swelling of Na5_1_* hydrogels render them a sponge-like behavior which might enhance the drug loading capacity [41]. Interestingly, as shown in Figure 5C, despite their high swelling ratio, the size of the hydrogels remained similar upon swelling in all the tested media.

The water retention kinetics of the Na5_1_* hydrogels are shown in Figure 5B. It can be observed that, after placing at 37 °C for 320 min, over 95% of their water content was evaporated and the hydrogels considerably reduced their volume (Figure 5C).

Additionally, the water-swollen hydrogels exhibited a lower water loss rate than those swollen in PBS or NaCl 0.9% (Figure 5B). The largest difference was noticed after 160 min, where the water-swollen hydrogels still had retained 42% of their initial water content, while the PBS- and NaCl-swollen hydrogels had only kept 27% and 25%, respectively. The faster evaporation of water in these hydrogels may indicate the presence of higher levels of free water in their structures since free water has the highest mobility and is the first to evaporate [42].

The macroscopic aspect of water-swollen hydrogels is shown in Figure 5C. It was observed that the visual characteristics of the hydrogel swollen in all the three tested media were similar, with all hydrogels showing a white color.

### 3.8. Hydrogels Loading and Release Ability

#### 3.8.1. Loading Na5_1_* Hydrogels with Caffeine

**Figure 6:**
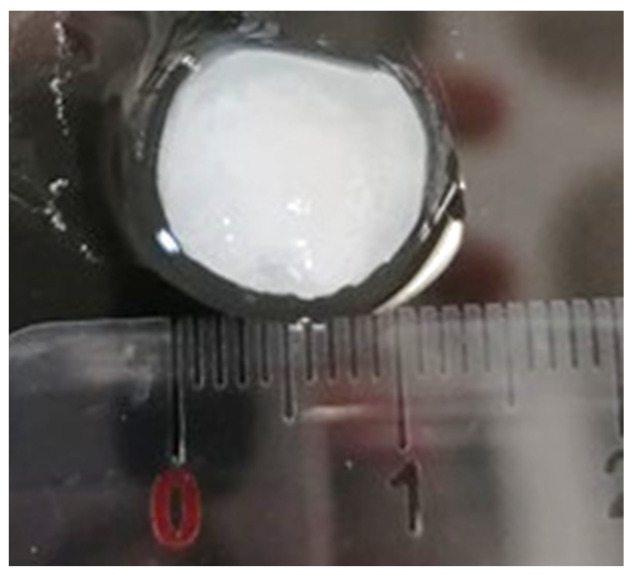
Macroscopic aspect of Na5_1_* hydrogels loaded with caffeine.

The Na5_1_* hydrogels were loaded with caffeine as a model drug (Figure 6). The procedure involved soaking the freeze-dried structures with a caffeine solution (1.0 wt%). The resulting loaded Na5_1_* hydrogels were opaque with a whitish color and their dimensions remained similar to those before soaking.

The entrapment efficiency of caffeine in the Na5_1_* hydrogels was found to be 5.82 ± 0.89%, with caffeine representing 1.02 ± 0.03% of the total weight of the hydrogel. This result is lower than the values reported in the literature for caffeine entrapment in cellulose-based hydrogel membranes (100%) [25] and β-glucans microparticles (96.52 ± 0.63%) [43]. This low EE% might be explained by the chemical structure of caffeine, that in water tends to protonate, and by the lower acetylation degree of the *N*-acetyl-glucosamine monomers of CGC. In fact, it was reported that the use of alkali solvent systems and freeze–thaw cycles promote the deacetylation of CGC chitin molecules [20]. The Na5_1_* hydrogels presented a degree of acetylation of 27.93 ± 2.82%, which indicates that chitin was converted into chitosan. Thus, the interactions between protonated caffeine and positively charged deacetylated chitin groups lead to repulsion due to similar charges. Similar behavior was reported for nanocarriers of chitosan where a caffeine entrapment efficiency of 17.25 ± 1.48% was observed [44].

#### 3.8.2. Characterization of the Na5_1_* Loaded Hydrogels

**Figure 7:**
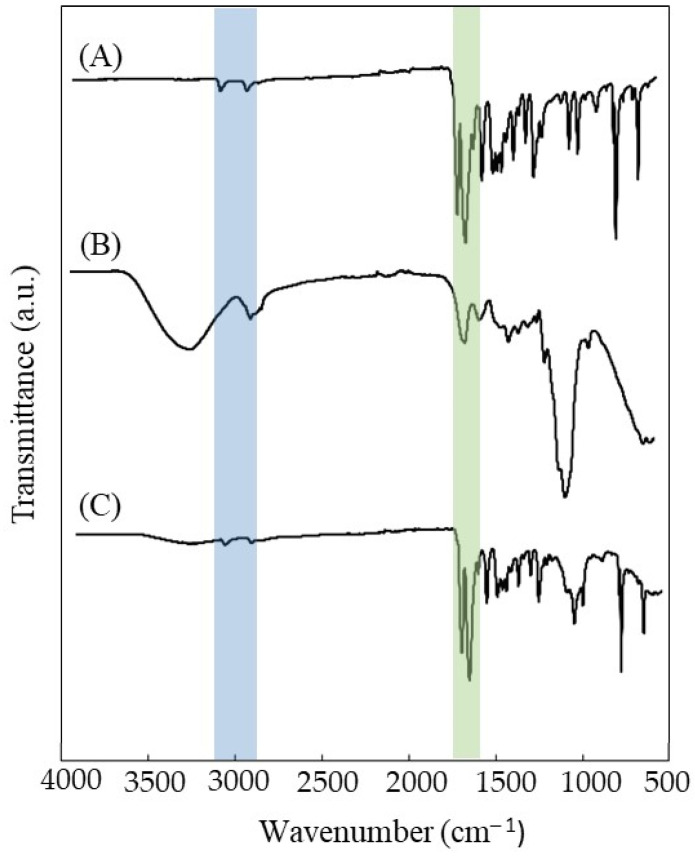
FTIR spectra of (**A**) caffeine, (**B**) Na5_1_* hydrogels, and (**C**) Na5_1_* loaded hydrogels.

The presence of caffeine in the Na5_1_* hydrogel was detected by FTIR analysis. Figure 7 shows the FTIR spectra of caffeine, the Na5_1_* hydrogel and the Na5_1_* loaded hydrogel. The caffeine spectrum (Figure 7A) displayed the typical bands of heterocyclic compounds, namely, at 3115 cm^−1^ and 2952 cm^−1^ which depict the stretching of C-H bonds. The absorption peaks at 1697 cm^−1^ and 1650 cm^−1^ are characteristic of the carbonyl group (C = O) of amide I and the additional adsorption peak at 1549 cm^−1^ can be attributed to amide II [45].

As expected, the Na5_1_* hydrogel spectrum (Figure 7B) is similar to previously reported CGC spectrum [20], presenting a characteristic a broad and intense band between 3000–3500 cm^−1^, typical of the O-H stretching of hydroxyl groups, which overlaps the stretching peaks of N-H. The C-H stretching corresponding to CH_3_ and CH_2_ appeared at wavenumbers 2919 and 2852 cm^−1^ respectively. The small peaks characteristics of β-1,3-glucans are noticed at 890, 1156, and 1370 cm^−1^ while β-1,6-glucans are represented by peaks at 922, 1045, and 1730 cm^−1^. The incorporation of caffeine in the hydrogel structure led to a general decrease in the intensity of Na5_1_* hydrogel spectrum bands, namely the O-H band around 3400 cm^−1^ and the C-O stretching of the saccharide structure at 1020 cm^−1^ (Figure 7C). This impact on the bands intensity might be explained by the high content of caffeine in the hydrogels structure (30.58 ± 4.28%, on a dry basis). Furthermore, peaks appearing at 3113 and 2955 cm^−1^ (heterocyclic compounds), 1697 and 1650 cm^−1^ (amide I) confirm the presence of caffeine, indicating a successful loading. Similar results were reported in several studies where caffeine was encapsulated in alginate beads [45] and chitosan nanoliposomes [46].

The effect of dehydration and the presence of caffeine on the mechanical and rheological properties of the Na5_1_* hydrogels is presented in Table 2. It can be seen that both the rehydration of the freeze-dried structure and its loading with caffeine apparently improved the mechanical parameters of the Na5_1_* hydrogels, in particular the compressive modulus, which increased significantly from 23.0 ± 0.89 to 38.06 ± 4.46 and 120.0 ± 61.64 kPa, respectively, thus demonstrating that caffeine increased the structure’s rigidity. Consequently, the rehydrated and the caffeine loaded hydrogels presented higher toughness (1.67 ± 0.09 and 1.8 ± 0.33 kJ/m^3^, respectively) and hardness (11.50 ± 0.58 and 15.6 ± 2.53 kPa, respectively) values than the original Na5_1_* hydrogel (0.78 ± 0.01 kJ/m^3^, 5.04 ± 0.14 kPa, respectively). These results suggest that the freeze-drying process reinforces the hydrogel structure, thus inducing a more rigid structure upon rehydratation. It is attributed to an increase of interactions (hydrogen bonds) between macromolecules upon drying, forming pore walls made of a more tightly packed and ordered hydrogen-bonded network structure, similarly to what is referred when producing polysaccharide films by solution casting and drying [47]. Loading caffeine into the same structures further strengthened the hydrogel’s network and endows enhanced mechanical properties. According to the literature [48], caffeine molecules are able to bind to saccharide molecules (e.g., glucose), which may promote the observed reinforcement of the hydrogel pore walls. Though, the nature of caffeine interactions with the polymeric CGC is likely to be more complex than simple hydrophobic biding. Further studies (e.g., NMR) would be needed to fully characterize CGC-caffeine bonds.

In the same way, the rheological properties of hydrogels were also improved by the presence of caffeine within the structure (Table 2, Figure 4B,C). The Na5_1_* rehydrated and the loaded hydrogels presented a rheological profile similar to the original Na5_1_* hydrogels, exhibiting a predominant elastic behavior (Figure 4B,C). This fact shows that rehydration of the freeze-dried hydrogels and the incorporation of caffeine into their structure had no significant impact on the viscoelastic degree of the structure and the crosslinked network remained homogenous [33]. As a solid-like structure, the storage moduli values were one order of magnitude higher than the loss moduli values (Figure 4). Nevertheless, both the rehydration process and caffeine loading improved the storage and the loss moduli, which is consistent with the observed enhanced mechanical properties. As demonstrated in Table 2, at a frequency of 1 Hz, G′ value was improved from 149.9 ± 9.8 kPa to 186.6 ± 22.0 and 315.0 ± 76.7 kPa and values of G″ increased from 11.9 ± 0.5 to 16.8 ± 2.5 and 29.3 kPa, for rehydrated and loaded hydrogels, respectively. The strengthening of hydrogels network of polycaprolactone-co-lactide (PCLA) by loading certain drugs into the structure was reported by Prince et al. [49]. Additional linkages promoted polymer-polymer interactions which increased G′ values and new polymer–drug interactions that raised G″ values.

#### 3.8.3. Release of Caffeine from the Na5_1_* Hydrogels

**Figure 8:**
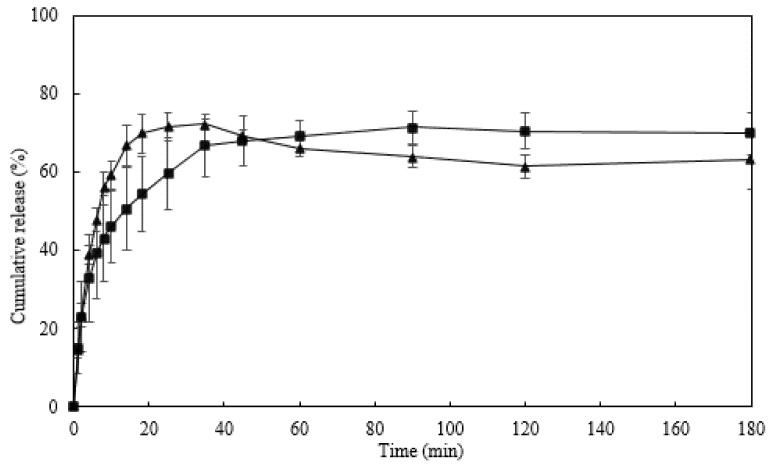
Caffeine release profile of Na5_1_* hydrogel in PBS (■) and NaCl 0.9% (▲), at 37 °C.

The caffeine release profile of the Na5_1_* loaded hydrogels was essayed in PBS (pH 7.4) and NaCl 0.9% (pH 5.5), at 37 °C (Figure 8). It can be observed that a similar caffeine release profile was obtained for both media, comprising an initial phase where caffeine was rapidly released (burst phase) followed by a second phase where a steady slower release was achieved. The initial burst can be explained by the release of caffeine loaded close to the surface of the hydrogel [50,51]. Moreover, the maximum caffeine released achieved was similar in both PBS and NaCl 0.9% solutions (71.4 ± 4.1% and 71.5 ± 3.5%, respectively), which might be explained by the similar swelling behavior of Na5_1_* hydrogels in each media (Figure 5A).

However, it can be noticed that the release of caffeine from the hydrogel was faster in NaCl 0.9% solution than in PBS solution. In the NaCl 0.9% solution, 50% of the loaded caffeine was release in the first 6 min, whereas in PBS solution it took 14 min to reach the same release. This behavior might be related to the difference in pH of the solutions that might have affected the solubility of caffeine [52]. Due to its alkalinity, caffeine solubility is enhanced by low pH values, which explains its rapid release rate in NaCl solution (pH 5.5). Additionally, the slightly higher ionic strength of the NaCl 0.9% solution promoted a faster release of caffeine due to decreased osmotic pressure within the hydrogel structure and weakened interactions between caffeine and polymer [53].

The mechanism of caffeine release from hydrogels is predominantly controlled by diffusion [54]. To evaluate the caffeine release kinetics from Na5_1_* loaded hydrogels, the first 60% of the released caffeine were analyzed using the Korsmeyer–Peppas model [26], according to the equation

Mt/M∞ = k tn(9)

where Mt and M∞ represent the amount of caffeine (g) released at time t and infinite time, respectively; k is the kinetic constant characteristic of the drug-polymer interaction and *n* is an empirical parameter for the release mechanism. According to this model, the diffusion mechanism can be classified as controlled diffusion (Fickian diffusion), anomalous transport (non-Fickian diffusion) and controlled swelling, as a function of the relationship between the diffusion rate and the polymer relaxation process [26,55]. For cylinder samples, a value of *n* ≤ 0.45 indicates a Fickian diffusion, 0.45 ≤ *n* ≤ 0.85 is a non-Fickian diffusion and *n* ≥ 0.85 represents a relaxation-controlled diffusion. Plotting ln (Mt/M∞) vs. ln (t), the kinetic parameters *n* and k can be calculated from the slope and the interception, respectively.

**Figure 9:**
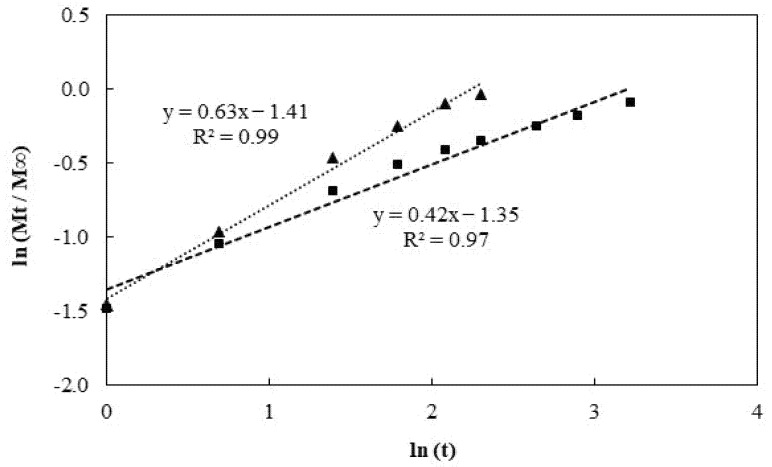
Plot of ln (Mt/M∞) vs. ln (t) for the caffeine release from Na5_1_* loaded hydrogels in PBS (■) and NaCl 0.9% (▲) solutions, following the Korsmeyer–Peppas model.

As shown in Figure 9, the kinetic parameters obtained in the PBS solution (*n* = 0.42 and k = 0.26) revealed a Fickian diffusion which indicates that caffeine diffusion through the hydrogel occurs slower than the relaxation of the polymer chains [55]. On the other hand, the release of caffeine in a NaCl 0.9% solution followed a non-Fickian diffusion, as *n* = 0.63 and k = 0.24. This mechanism suggests that the drug diffusion rate and the polymer relaxation process are relevant to the drug release rate [33]. The results obtained revealed that drug release from Na5_1_* hydrogels is not only dependent on the physicochemical properties of the loaded drug, but also on the properties of the medium used to release the drug.

## 4. Conclusions

The procedure for preparing CGC hydrogels comprising polymer dissolution in NaOH by the freeze–thaw method, followed by coagulation by dialysis of the obtained aqueous solution, was optimized by reducing the number of freeze–thaw cycles and the total freezing time. The optimized methodology resulted in CGC hydrogels with improved rheological, mechanical, and swelling properties, which were assayed for their ability for caffeine loading. The loaded hydrogels displayed improved mechanical and rheological properties, with caffeine release profiles following a Fickian diffusion mechanism in the PBS solution and a non-Fickian diffusion in the NaCl 0.9% solution. Therefore, this study demonstrated that CGC can be processed into hydrogels by a simple procedure and the resulting structures possess suitable properties for their use as drug delivery systems.

## Figures and Tables

**Table 1:** Chemical characterization of CGC hydrogels obtained by different number of freeze–thaw cycles: 1 cycle (Na5_1_ hydrogel), 2 cycles (Na5_2_ hydrogel), 3 cycles (Na5_3_ hydrogel) and 1 cycle with reduced freezing time (Na5_1_* hydrogel); n.a., data not available.

Samples	Na5_1_	Na5_2_	Na5_3_	Na5_1_*	Na5 [22]
No. of cycles	1	2	3	1	4
Freezing time (h)	48	48	48	18	48
Polymer content (wt%)	1.68 ± 0.17	1.58 ± 0.04	1.42 ± 0.02	1.66 ± 0.11	2.28
Water content (wt%)	98.22 ± 0.20	98.45 ± 0.06	98.58 ± 0.02	97.63 ± 0.12	97.72
Chitin content (%)	25.63 ± 0.78	24.71 ± 2.98	23.85 ± 0.14	21.51 ± 1.49	n.a.

**Table 2:** Effect of caffeine on the mechanical and rheological properties of the Na5_1_* hydrogel.

	Sample	Na5_1_* Hydrogel	Na5_1_* Rehydrated Hydrogel	Na5_1_* Loaded Hydrogel
Mechanical properties	Compressive modulus (kPa)	23.0 ± 0.89	38.06 ± 4.46	120.0 ± 61.64
	Toughness (kJ/m^3^)	0.78 ± 0.01	1.67 ± 0.09	1.8 ± 0.33
	Hardness (kPa)	5.04 ± 0.14	11.50 ± 0.58	15.6 ± 2.53
Rheological properties	Storage modulus_1 Hz_ (G′, kPa)	149.9 ± 9.8	186.8 ± 22.0	315.0 ± 76.7
	Loss modulus_1 Hz_ (G″, kPa)	11.9 ± 0.5	16.8 ± 2.5	29.3 ± 8.4

## Data Availability

Data will be made available upon request.
